# Correlations between lipid profiles and atherosclerotic plaque characteristics in adult patients with type 2 diabetes mellitus

**DOI:** 10.3389/fcdhc.2025.1688715

**Published:** 2025-12-01

**Authors:** Lili Qiao, Jiameng Miao, Weixuan Du, Yi Liu, Yanrong Chen, Kaiyue Xu, Chen Wang, Heye Chen

**Affiliations:** 1 Department of Endocrinology, Affiliated Hospital of Hebei University, Baoding, Hebei, China; 2 Department of Diabetic Foot Care, 82nd Group Army Hospital, Baoding, Hebei, China; 3 Education Department, Affiliated Hospital of Hebei University, Baoding, Hebei, China; 4 First Department of Endocrinology, Tangshan Worker’s Hospital, Tangshan, Hebei, China; 5 Department of Endocrinology, Jinhua Central Hospital Department of Endocrinology, Jinhua, Zhejiang, China

**Keywords:** type 2 diabetes mellitus, lipids, atherosclerosis, hypoechoic plaques, unstable plaque

## Abstract

**Background and purpose:**

Diabetes mellitus and dyslipidemia are major risk factors for atherosclerosis. Hypoechoic plaques, which indicate vulnerable or unstable plaques, may rupture and lead to ischemic stroke, cognitive impairment, increased adverse cardiac events, and even death. This study aimed to investigate the correlation between plasma lipid levels and the characteristics of atherosclerotic plaques in adult patients with type 2 diabetes mellitus.

**Methods:**

A retrospective analysis was conducted on adult patients with type 2 mellitus who were hospitalized in the Department of Endocrinology at Affiliated Hospital of Hebei University between January 2017 and December 2021.Patients were categorized into two groups based on arterial ultrasound results. Statistical analyses were performed to compare plasma lipid levels and plaque characteristics across the groups.

**Results:**

1) Statistically significant differences were observed among the two groups in terms of gender, hypertension, age, duration of diabetes mellitus, plaque location, triglycerides (TG),total cholesterol (TC), Apolipoprotein A1 (Apo A1),very-low-density lipoprotein (VLDL), VLDL/apolipoprotein B(ApoB), high-density lipoprotein cholesterol (HDL)/ApoA1 (*P*<0.05). 2) Univariate and multivariate regression analyses revealed that VLDL,VLDL/ApoB and HDL/ApoA1 were correlated with plaque stability, the higher the levels of VLDL,VLDL/ApoB and HDL/ApoA1,the more likely they were to be hypoechoic plaque group (OR>1, *P*<0.05). 3) VLDL, VLDL/ApoB and HDL/ApoA1 showed predictive value for determining whether patients with type 2 diabetes had stable plaques. The area under the receiver operating characteristic (ROC) curve for VLDL, VLDL/ApoB and HDL/ApoA1 respectively were 0.789,0.779 and 0.728.

**Conclusion:**

In clinical practice, the characteristics of atherosclerotic plaques and lipid profiles should be jointly evaluated to guide targeted treatment and effectively reduce the risk of atherosclerotic cardiovascular disease.

## 1 Introduction

Diabetes is a group of metabolic diseases characterized by hyperglycemia (1). Type 2 diabetes mellitus (T2DM) accounts for 90-95% of all diabetes cases (2). The global prevalence of diabetes has increased considerably over the past 30 years (3). Between 1990 and 2022,the number of adults aged ≥18 with diabetes worldwide rose from approximately 200 million to 828 million (3). In 2022,the number of adult patients with diabetes in China reached approximately 148 million, accounting for 18% of the global total (3). The incidence and treatment costs of diabetes continue to rise, compounded by the serious health threats posed by diabetes and its complications (4, 5). This situation imposes a substantial economic burden on both individuals and society (6), making diabetes a global public health challenge (4, 5). It is estimated that by 2030,global medical expenses related to diabetes will reach United States Dollar (USD) 1 trillion, with China alone spending USD 165.3 billion on diabetes care in 2022 (7). Data from IQVIA indicate that in China,84.6% of diabetes-related medical expenses are attributed to complications. These complications often manifest at the vascular level, including endothelial dysfunction and atherosclerosis (AS) (8). The clinical manifestations of AS typically involve the coronary arteries, lower extremities, and extracranial carotid arteries (9, 10). Patients with T2DM tend to develop atherosclerotic cardiovascular disease (ASCVD) earlier (11), with greater severity and more widespread lesion distribution compared to those without diabetes (12). Furthermore, the risk of cardiovascular disease (CVD) and hospitalization for heart failure in patients with diabetes is 2–4 times (13) and 2 times (14, 15) higher, respectively, than in those without diabetes. Compared to non-diabetic patients, those with diabetes have higher rates of intermittent claudication (26.3% versus 15.3%) and higher incidence (5. 1% versus 2. 1%) (16). A multicenter cross-sectional study found that the prevalence of peripheral arterial disease (PAD) among patients with diabetes >70 years reached 71% (17). The leading cause of death in patients with diabetes is ASCVD (18), with coronary heart disease (CHD) accounting for approximately 40%, heart failure for 15%, and stroke for 10% (19).

While recent data indicate a notable decline in ASCVD incidence among people living with T2DM (20), T2DM itself continues to be an extremely common condition and a key risk factor for ASCVD (21, 22), particularly when accompanied by dyslipidemia, commonly referred to as atherosclerotic dyslipidemia (23). This condition is characterized by elevated triglycerides (TG),the presence of small, dense low-density lipoprotein particles (sdLDL) (24), and reduced levels of high-density lipoprotein (HDL). A cross-sectional study involving 4,807 patients with T2DM from endocrinology outpatient departments in 20 tertiary hospitals across major Chinese cities reported a dyslipidemia prevalence of 67. 1% (25). The lipid profile of patients with T2DM often exhibits mixed dyslipidemia, including both fasting and postprandial hypertriglyceridemia, reduced HDL levels, normal or slightly elevated levels of total cholesterol (TC) and low-density lipoprotein (LDL), and an increased proportion of sdLDL particles (26). Epidemiological, genetic, and clinical intervention studies have confirmed that LDL is a key pathogenic factor in ASCVD (27). Therefore, multiple clinical guidelines recommend lowering LDL levels to reduce cardiovascular risk (24, 28, 29). Recent evidence also highlights the atherogenic roles of other apolipoprotein B (ApoB)-containing lipoproteins, such as triglyceride-rich lipoproteins (TRLs), their remnants, and lipoprotein(a) (Lp[a]), in the pathophysiology of ASCVD (27). Given the complex lipid abnormalities in patients with T2DM, reliance on LDL alone may underestimate risk. The latest European Society of Cardiology (ESC) Guidelines on Lipid Disorders states: Lipoprotein(a) [Lp(a)] increases the risk of ASCVD and aortic valve stenosis (AVS); for patients with T2DM without ASCVD, the SCORE2-Diabetes model is recommended to assess the 10-year risk of CVD (30). In addition, both the SCORE2 and SCORE2-OP models use non-HDL (TC minus HDL) as the input indicator (30). Another important development in lipidology is the renewed recognition of plasma TG (31) and residual cholesterol (RC) (24, 26) as both risk factors and potential causal agents of ASCVD.

Carotid plaque is an indicator of AS and is associated with the future risk of ASCVD (32). Multiple guidelines recommend that the presence of carotid plaques be considered equivalent to the risk of ASCVD (33, 34). Carotid artery ultrasound can detect the active components of carotid plaques. Hypoechoic plaques indicate vulnerable or unstable plaques, which may lead to plaque rupture and cause ischemic stroke events (35–37), cognitive impairment (38), increased adverse cardiac events (39–42), and even death (43–45).

This study aimed to explore the contribution of different lipid components to the development of AS and their impact on plaque properties in adult patients with T2DM.

## 2 Materials and methods

### 2.1 Research participants

This was a retrospective analysis of adult patients with T2DM who were treated at the Department of Endocrinology, Affiliated Hospital of Hebei University, from January 2017 to December 2021.

Inclusion criteria: Patients who met the World Health Organization (WHO) diagnostic criteria for diabetes; underwent bilateral carotid artery color Doppler ultrasound; and had complete clinical data, including general information medical history, blood biochemical measurements, and ultrasound results.
Exclusion criteria: Undefined type of diabetes or non-type 2 diabetes; acute diabetic complications; severe liver or kidney dysfunction; malnutrition; immune system diseases; thyroid dysfunction; familial hypercholesterolemia; hemoglobinopathy; major psychiatric disorders; acute cardiovascular or cerebrovascular disease; mental illness or impaired consciousness; active infection; stress conditions; and other malignancies.

### 2.2 Research methods

Eligible adult patients with a confirmed diagnosis of T2DM were included as the observation group. General information (age, sex, height, weight),smoking history, alcohol history, hypertension, *duration of diabetes mellitus* (it is defined as the period from the diagnosis of T2DM to the current hospitalization, with the unit: years.),fasting plasma glucose, glycated hemoglobin, homocysteine, carotid ultrasound findings, and blood lipid parameters (including TG, TC, LDL, HDL, very-low-density lipoprotein (VLDL),apolipoprotein A1 (ApoA1), and apolipoprotein B (ApoB), were collected, meanwhile, information on drug treatment was collected, including the usage of statin therapy, antiplatelet treatment, and hypoglycemic agents (including metformin, glucagon-like peptide-1 receptor agonists [GLP-1 RAs], and sodium-glucose cotransporter 2 inhibitors [SGLT2is]).Differences in plasma lipid components among the groups were analyzed to explore the correlation between lipid levels and plaque characteristics, and to evaluate the predictive value of each variable for plaque stability.

Blood lipid measurements were performed using an instrument from Beckman Coulter (United States). LDL was measured by the direct catalase clearance method, and VLDL was calculated using a standard formula.

Technical details of carotid artery color Doppler ultrasound examination

A Philips EPIQ 7C ultrasound diagnostic system with a 7.5–10 MHz probe was used. Patients were placed in a supine position, with their heads turned to the opposite side. Continuous transverse and longitudinal scans were performed from the origin of the common carotid artery to the siphon segment of the internal carotid artery. The location, size, and echo type of plaques were recorded. Ultrasound results were interpreted in a double-blinded manner by 2 trained radiologists. In case of inconsistent opinions, a consensus was reached through discussion.

Routine ultrasound examination was performed in this study. The reporting method for the echo intensity of carotid plaques adopts the modified Gray-Weale classification into five levels of echogenicity, with types 1–4 based on the proportion of hypoechoic (darker) to hyperechoic (brighter) tissue, and a type 5 for plaques that are difficult to classify due to heavy calcification (type 1: uniform hypoechoic; type 2: more than 50% hypoechoic; type 3: less than 50% hypoechoic; type 4:uniform hyperechoic; type 5: heavy calcification).Per this classification system, plaques of Type 1 and Type 2—predominantly hypoechoic—were designated as Group A1 in this study, while plaques of Type 3 to Type 5 were designated as Group A0.

### 2.3 Definition and formula

Diagnosis of diabetes (46): Fasting plasma glucose ≥126 mg/dL (7.0 mmol/L); 2-h plasma glucose ≥200 mg/dL (11.1 mmol/L) after a 75-g oral glucose tolerance test; glycated hemoglobin ≥6.5% (48 mmol/mol); or random plasma glucose ≥200 mg/dL (11.1 mmol/L),confirmed by repeat testing in the absence of symptoms of hyperglycemia or hyperglycemic crisis.

Carotid plaque is defined as (47) “a local structure that invades at least 0.5 mm into the arterial lumen, exceeds 50% of the surrounding intima-media thickness, or has a thickness ≥1.5 mm. Body Mass Index (BMI): Calculated as weight in kilograms divided by the square of height in meters (kg/m²).

In this study, VLDL was calculated. The VLDL calculation formula: VLDL-C (mmol/L) = TC – LDL – HDL.

### 2.4 Statistical methods

Statistical analysis and graphing were performed using SPSS 25.0 and RStudio (R4.4.0). Categorical data were analyzed using the chi-square test or Fisher’s exact test, expressed as number of cases (percentage). Continuous data conforming to normal distribution and homogeneity of variance between two groups were analyzed using the t-test and presented as mean ± standard deviation. Continuous data violating these assumptions were analyzed using the Mann-Whitney U test and expressed as median [interquartile range]. Regression analysis included both univariate and multivariate logistic regression. Restricted cubic spline (RCS) analysis was employed to visualize the nonlinear relationships between VLDL,VLDL/ApoB, HDL/ApoA1 and group classification. Receiver Operating Characteristic (ROC) curves were plotted, and the Area Under the ROC Curve (AUC) was calculated to evaluate the predictive value of VLDL,VLDL/ApoB, and HDL/ApoA1.A *P* -value < 0.05 was considered statistically significant.

## 3 Results

### 3.1 Clinical characteristics of study participants

A total of 199 patients with T2DM were included. These patients were further divided into two subgroups based on carotid ultrasound results: Group A0, patients with diabetes and AS with hyperechoic plaques; Group A1, patients with diabetes and AS with hypoechoic plaques. **Table 1** presents the clinical characteristics of the two groups. Chi-square tests were conducted for gender, smoking history, alcohol history, and hypertension in groups 0 and 1; t-tests were conducted for age and Apo A1;and Mann-Whitney U tests were applied for the remaining indicators. The results revealed significant differences (*P*<0.05) between the two groups in gender, hypertension, age, duration of diabetes mellitus, TG, TC, ApoA1, VLDL, VLDL/ApoB, and HDL/ApoA1.

**Table 1:** Baseline characteristics of the study participants.

Indicator		Total (N= 199)	Group0 (N=78)	Group1 (N= 121)	χ2/t/z	*P*
Sex	M	124 (62.31%)	41 (52.56%)	83 (68.60%)	5.190	0.023
	F	75 (37.69%)	37 (47.44%)	38 (31.40%)		
Smoking History	N	167 (83.92%)	68 (87. 18%)	99 (81.82%)	1.010	0.315
	Y	32 (16.08%)	10 (12.82%)	22 (18. 18%)		
Alcohol History	N	162 (81.41%)	62 (79.49%)	100 (82.64%)	0.312	0.576
	Y	37 (18.59%)	16 (20.51%)	21 (17.36%)		
Hypertension	N	89 (44.72%)	43 (55. 13%)	46 (38.02%)	5.617	0.018
	Y	110 (55.28%)	35 (44.87%)	75 (61.98%)		
drug treatment	statin therapy	45 (22.61%)	20 (25.93%)	25 (20.66%)	0.672	0.412
	antiplatelet treatment	45 (22.61%)	20 (25.93%)	25 (20.66%)	0.672	0.412
	GLP-1 RAs/SGLT-2is	3 (1.51%)	0	3 (2.48%)	2.613	0.105
	metformin	95 (47.74%)	38 (48.15%)	57 (47.11%)	0.049	0.824
Age (years)		50.06±14.30	57. 13±13.09	45.50±13.19	6.087	<0.001
Duration of diabetes mellitus (years)		10.00 [4.58;16.08]	11.62 [6.67;16.92]	7.50 [3.42;15.33]	-2.820	0.005
BMI (kg/m^2^)		25.95 [23.44;28.75]	26.23 [23.24;28.62]	25.95 [24.03;28.77]	-0.395	0.693
plaque location	*external carotid artery*	6 (3.01%)	2 (2.56%)	3 (2.48%)	0.001	0.97
	*common carotid artery*	79 (39.70%)	14 (17.95%)	90 (74.38%)	60.537	<0.001
	*carotid bulb*	30 (15.08%)	14 (17.95%)	14 (11.57%)	1.596	0.206
	*internal carotid artery*	84 (42.21%)	48 (61.54%)	14 (11.57%)	52.936	<0.001
Fasting Plasma Glucose (mmol/L)		9.20 [6.90;12.50]	8.90 [6.93;11.95]	9.70 [6.90;12.80]	-0.680	0.497
Glycated Hemoglobin (%)		8.80 [7.30;10.30]	8.85 [7.53;10.00]	8.80 [7. 10;10.50]	-0.032	0.975
Homocysteine (umol/L)		15.00 [13.00;20.00]	15.00 [12.00;19.00]	15.00 [13.00;21.00]	-1.448	0.148
TG (mmol/L)		1.64 [1. 10;2.36]	1.48 [0.98;2.06]	1.79 [1. 17;2.63]	-2.378	0.017
TC (mmol/L)		4.58 [4.08;5.26]	4.40 [3.65;5.03]	4.77 [4.24;5.33]	-2.704	0.007
HDL (mmol/L)		1.04 [0.90;1.22]	1.05 [0.90;1. 15]	1.03 [0.90;1.27]	-0.802	0.423
LDL (mmol/L)		2.98 [2.51;3.50]	2.95 [2.40;3.56]	3.06 [2.58;3.46]	-0.813	0.416
Apo A1 (g/L)		1. 17±0.25	1.24±0.22	1. 12±0.27	3.161	0.002
ApoB (g/L)		0.92 [0.78;1. 10]	0.92 [0.78;1.08]	0.92 [0.78;1. 10]	-0.072	0.943
VLDL (mmol/L)		0.51 [0.38;0.70]	0.39 [0.33;0.49]	0.62 [0.47;0.77]	-6.871	<0.001
VLDL/ApoB		0.22 [0. 18;0.30]	0.19 [0. 13;0.21]	0.26 [0.21;0.34]	-6.646	<0.001
HDL/ApoA1		0.35 [0.31;0.39]	0.33 [0.30;0.37]	0.37 [0.33;0.42]	-5.431	<0.001

M, man; F, female; N, no; Y, yes; BMI, body mass index; TC, total cholesterol; TG, triglycerides; HDL, high-density lipoprotein cholesterol; LDL, low-density lipoprotein; VLDL, very-low-density lipoprotein; ApoA1, apolipoprotein A1; ApoB, apolipoproteinB.

Characteristics of High-Risk Population with Hypoechoic Plaques

In Group A1 (hypoechoic plaques), the proportion of males was higher (68.60% vs. 52.56% in Group A0), the prevalence of hypertension was higher (61.98% vs. 44.87% in Group A0), the age was younger (45.50±13.19 years vs. 57.13±13.09 years in Group A0), and the duration of diabetes was shorter (7.50 [3.42; 15.33] years vs. 11.62 [6.67; 16.92] years in Group A0). Additionally, Group A1 showed higher levels of TG (1.79 [1.17; 2.63] mmol/L), TC (4.77 [4.24; 5.33] mmol/L), and VLDL (0.62 [0.47; 0.77] mmol/L), as well as higher ratios of VLDL/ApoB(0.26 [0.21; 0.34]) and HDL/ApoA1(0.37 [0.33; 0.42]) (all *P*<0.05). These results suggest that patients with T2DM who are young, male, complicated with hypertension, have a short duration of diabetes, and present with the aforementioned abnormal lipid indices are a population at high risk of hypoechoic plaques.

Chi-square test and Fisher’s exact test were used to analyze medication use and plaque location between the two groups (**Table 1**). The results showed a significant difference in plaque location between the two groups (*P*<0.05): hypoechoic plaques were more likely to occur in the common carotid artery, while hyperechoic plaques were more likely to occur in the internal carotid artery. However, there was no statistically significant difference in drug treatment between the two groups.

### 3.2 Correlation analysis between variables and plaque properties

Continuous variables VLDL,VLDL/ApoB, and HDL/ApoA1 were divided into quartiles Q1 -Q4, with the first quartile Q1 as the reference. Model 1 presents the results without adjustment for any variables. Model 2 adjusts for gender, hypertension, age, duration of diabetes mellitus, and TG. Model 3 further adjusts for smoking history, alcohol consumption history, BMI, fasting plasma glucose, glycated hemoglobin, and homocysteine based on Model 2.

As shown in **Table 2**, the results indicate that across Model 1, Model 2, and Model 3, higher levels of VLDL,VLDL/ApoB, and HDL/ApoA1 were associated with a tendency toward the hypoechoic plaque group (OR > 1, *P* < 0.05).

**Table 2:** Univariate and multivariate regression analysis.

Item	Model 1		Model 2		Model 3	
	OR(95%CI)	*P*	OR(95%CI)	*P*	OR(95%CI)	*P*
VLDL						
Q1	Reference		Reference		Reference	
Q2	1.154 (0.517, 2.574)	0.727	1.233 (0.503, 3.020)	0.647	1.198 (0.475, 3.026)	0.702
Q3	5.383 (2.296, 12.623)	<0.001	3.670 (1.398, 9.634)	0.008	4.174 (1.505, 11.580)	0.006
Q4	18.700 (5.844, 59.835)	<0.001	16.404 (4.472, 60.165)	<0.001	17.027 (4.499, 64.447)	<0.001
VLDL/ApoB						
Q1	Reference		Reference		Reference	
Q2	1.185 (0.528, 2.660)	0.680	1.308 (0.527, 3.248)	0.563	1.334 (0.505, 3.525)	0.561
Q3	7.901 (3.132, 19.933)	<0.001	5.568 (1.999, 15.512)	0.001	6.032 (2.054, 17.717)	0.001
Q4	10.921 (4.075, 29.263)	<0.001	10.072 (3.279, 30.934)	<0.001	11.214 (3.396, 37.033)	<0.001
HDL/ApoA						
Q1	Reference		Reference		Reference	
Q2	2.667 (1.188, 5.985)	0.017	3.607 (1.383, 9.403)	0.009	4.364 (1.574, 12.099)	0.005
Q3	2.807 (1.243, 6.339)	0.013	2.396 (0.935, 6.143)	0.069	2.396 (0.894, 6.422)	0.082
Q4	10.921 (4.075, 29.263)	<0.001	17.545 (5.204, 59.154)	<0.001	21.713 (6.012, 78.409)	<0.001

Model 1: without adjustment.

Model 2: adjusts for gender, hypertension, age, duration of diabetes mellitus, and TG.

Model 3: adjusts for smoking history, alcohol consumption history, BMI, fasting plasma glucose, glycated hemoglobin, and homocysteine based on Model 2.

**Figure 1:**
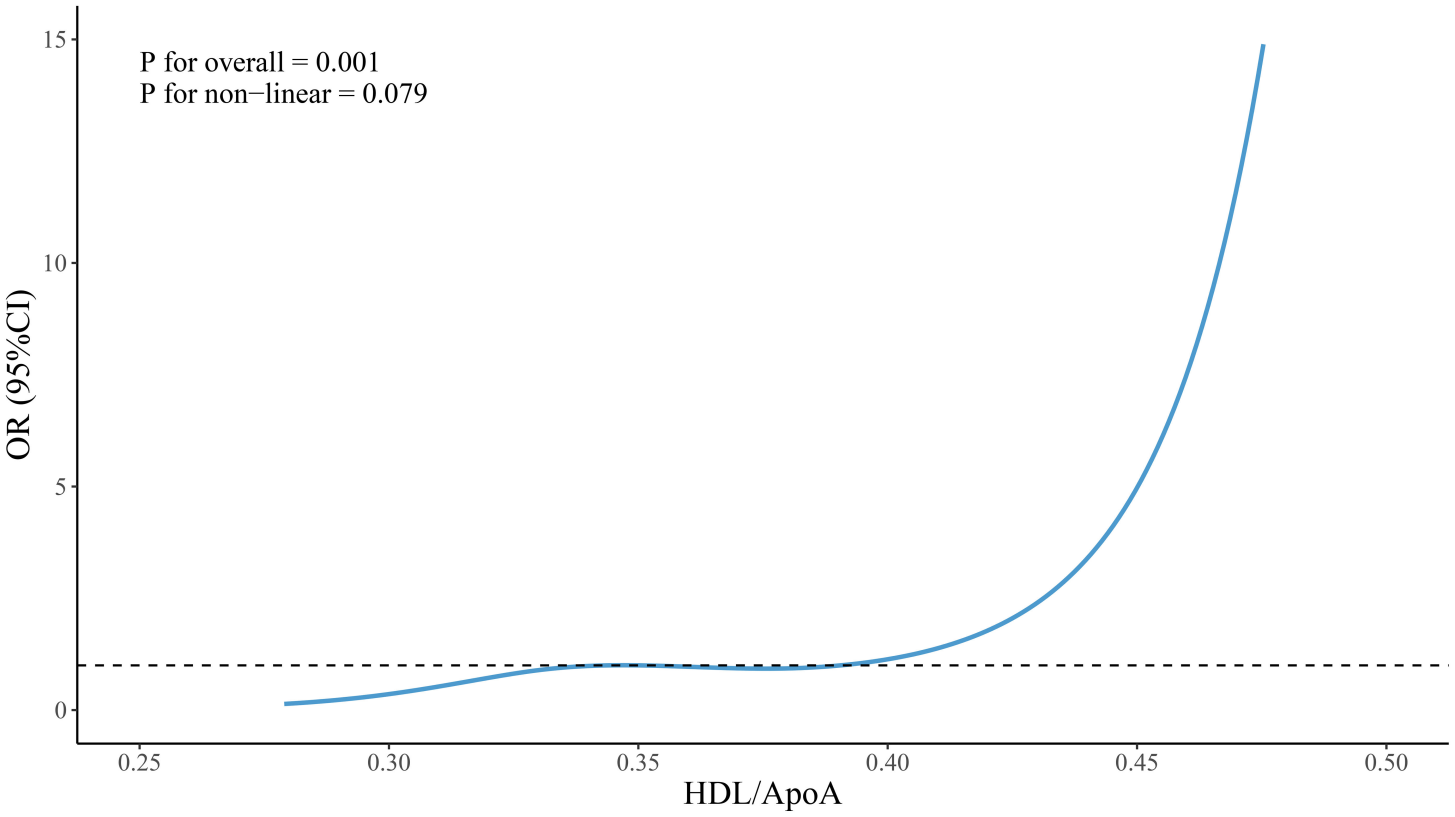
Nonlinear associations between HDL/ApoA and groupings.

**Figure 2:**
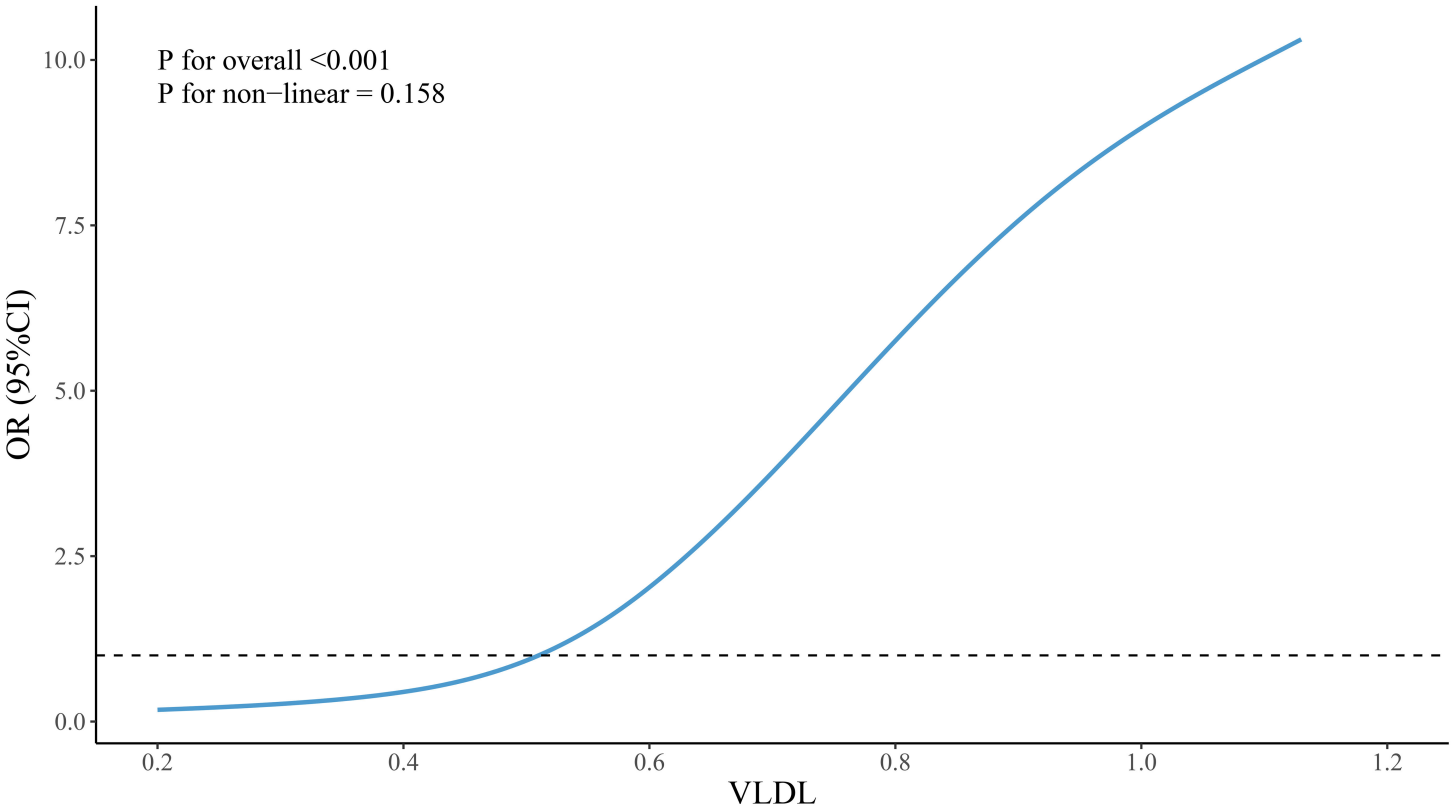
Nonlinear associations between VLDL and groupings.

**Figure 3:**
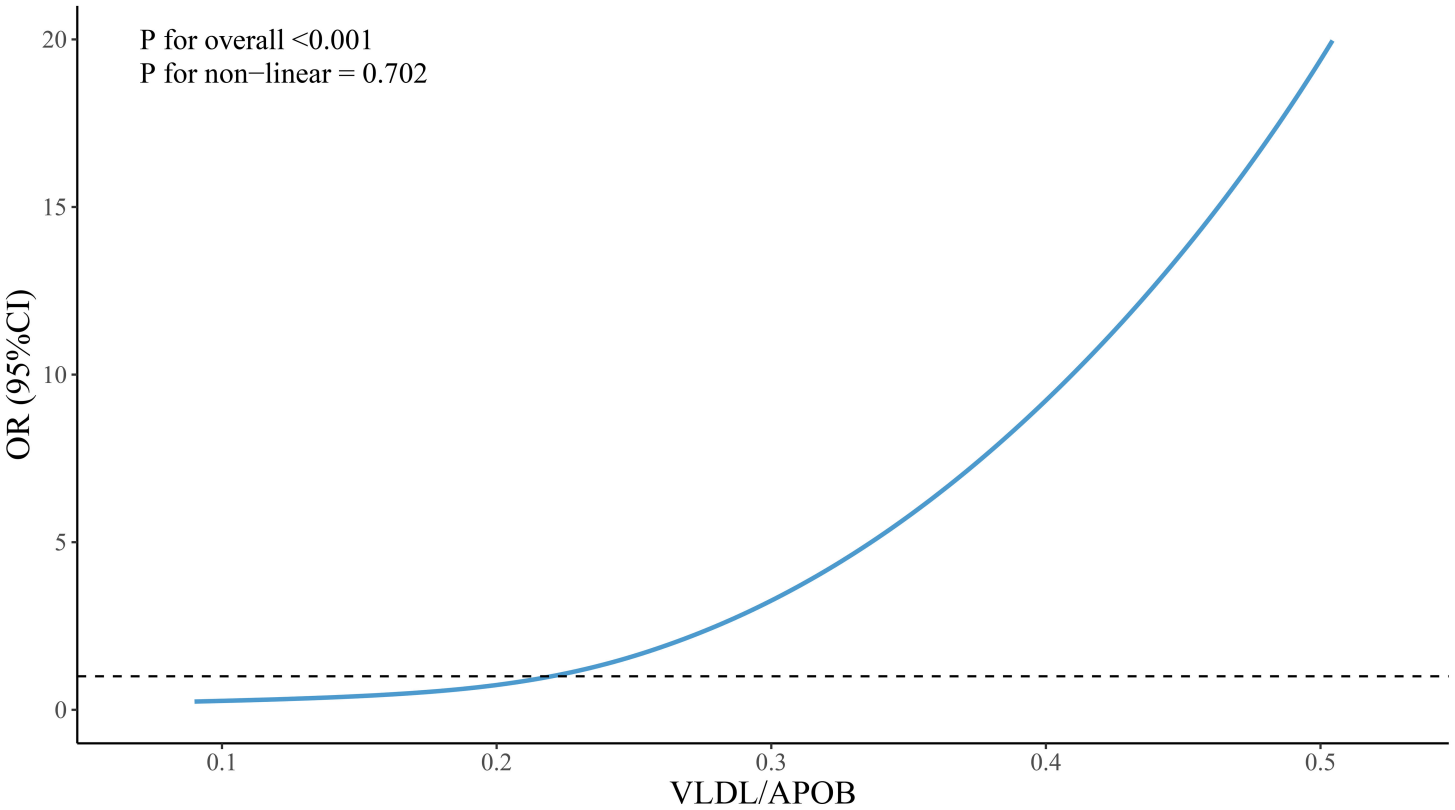
Nonlinear associations between VLDL/ApoB and groupings.

Restricted cubic spline (RCS) analysis was employed to visualize the nonlinear relationships between VLDL, VLDL/ApoB, HDL/ApoA1 and the grouping variable. The results, as depicted in **Figures 1**–**3**, revealed no significant nonlinear relationships between VLDL, VLDL/ApoB, HDL/ApoA1 and the grouping variable(*P* > 0.05).

### 3.3 Assessment of the ability of positive to evaluate carotid artery plaques

**Figure 4:**
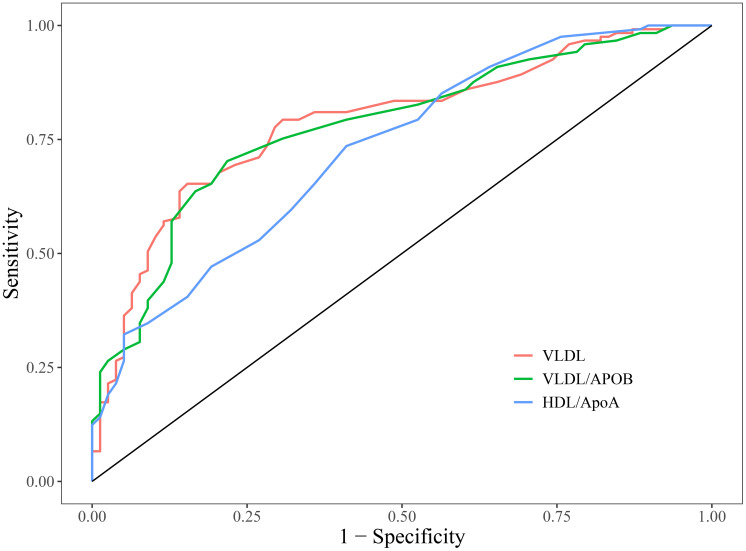
ROC curves of the positive indicators.

An ROC curve was drawn, and the AUC was calculated to evaluate the predictive value of lipid indicators for stable plaques. **Table 3**, **Figure 4** present the predictive value of VLDL,VLDL/ApoB, and HDL/ApoA1 for determining whether T2DM is associated with stable plaques. The results demonstrated that VLDL yielded an AUC of 0.789 (cut-off value: 0.55;sensitivity: 0.653; specificity: 0.846), VLDL/ApoB showed an AUC of 0.779 (cut-off:0.22; sensitivity:0.702; specificity:0.782), and HDL/ApoA1 achieved an AUC of 0.728 (cut-off: 0.34; sensitivity: 0.736; specificity: 0.590). These findings indicate that VLDL, VLDL/ApoB, and HDL/ApoA1 all possess good predictive and diagnostic value for Group 1.

**Table 3:** Assessment of the ability of positive indicators to evaluate carotid artery plaques.

Item	AUC	cut-off value	sensitivity	specificity	95%CI	*P*
VLDL	0.789	0.55	0.653	0.846	0.725 0.852	<0.001
VLDL/ApoB	0.779	0.22	0.702	0.782	0.714 0.844	<0.001
HDL/ApoA1	0.728	0.34	0.736	0.590	0.657 0.798	<0.001

## 4 Discussion

Carotid plaque is an indicator of AS, and its morphological characteristics (such as hypoechoic features) are associated with adverse clinical outcomes (35–45). One study found that hypoechoic plaques are independent predictors of acute coronary syndrome, with a hazard ratio as high as 22.8 (*P*<0.001) (48). The current practice guideline recommends carotid US for the detection of atherosclerotic plaques and for assessing of the atherosclerotic burden with a class IIa indication (49). Risk scores developed for the general population are not recommended for CV risk assessment in patients with T2DM. Practically, patients with T2DM have at least a moderate 10-year risk of fatal CVD. Assessment of carotid plaque burden on carotid US is considered as a risk modifier in individuals with moderate or low risk but may also influence the targets, treatment and follow-up in high and very high risk individuals (30). The composition of a plaque, as determined by its features on ultrasound, can help predict the risk of future cardiovascular events and stroke, especially when used with other clinical factors. Although previous studies have primarily focused on the correlation between blood lipid levels and AS, fewer have assessed the relationship between lipid indicators and plaque characteristics in adult patients with T2DM, as well as their predictive value for plaque stability. In this study, we analyzed the relationship between various blood lipid indicators and atherosclerotic plaque characteristics in adult patients with T2DM,along with their predictive value for plaque stability. The conclusions are as follows: 1) Statistically significant differences were observed among the two groups in terms of gender, hypertension, age, duration of diabetes mellitus, plaque location, TG, TC, Apo A1, VLDL, VLDL/ApoB, HDL/ApoA1 (*P*<0.05). 2) Univariate and multivariate regression analyses revealed that VLDL, VLDL/ApoB, and HDL/ApoA1 were associated with plaque stability. Elevated levels of VLDL, VLDL/ApoB and HDL/ApoA1 were identified as risk factors for the presence of unstable plaques. 3 VLDL,VLDL/ApoB and HDL/ApoA1 showed predictive value for determining whether patients with type 2 diabetes had stable plaques. The area under the receiver operating characteristic (ROC) curve for VLDL,VLDL/ApoB and HDL/ApoA1 respectively were 0.789,0.779 and 0.728. In this study, hypoechoic plaques were more likely to occur in the common carotid artery, while hyperechoic plaques were more likely to occur in the internal carotid artery. The possible reason is that the hemodynamics at the bulb of the common carotid artery are complex, and the shear stress on the vascular wall is uneven, which is prone to cause endothelial damage, promote lipid deposition and inflammatory response, and thus lead to plaque formation and instability (50).

Although LDL is a strong predictor of ASCVD (51, 52), patients with T2DM typically do not exhibit significantly elevated LDL levels (53, 54). Several studies have confirmed that even with statin-induced control of LDL levels, a high residual risk of CVD persists (55, 56). A case-control study by Puig et al. found that, compared with the control group, patients in the hypoechoic plaque and acute stroke groups had significantly lower TC, LDL and ApoA-1 levels (57). Furthermore, an autopsy study of sudden coronary death in men revealed that plaque rupture was strongly associated with low HDL levels (58). It is well known that HDL is inversely correlated with ASCVD. ApoA1 is the main protein component of HDL particles (accounting for 292 approximately 65%~75%),while other lipoproteins contain very little ApoA1.Therefore,serum ApoA1 levels reflect HDL particle levels and are significantly positively correlated with HDL. This study confirms that HDL/ApoA1—rather than TC, LDL, ApoA-1, or HDL—holds predictive value for plaque stability: the higher this ratio, the more unstable the atherosclerotic plaque. This may stem from differences between the study population and those in previous research. In patients with T2DM,the quantity, composition, and function of HDL are significantly affected (59).Extending the lipid panel with VLDL, Apo B and ApoA1 from the first evaluation of a patient with T2DM may indicate performing an initial carotid US evaluation and further carotid US monitoring.

Residual cholesterol (RC) is a concept proposed by the Nordestgaard team, specifically referring to the cholesterol contained in triglyceride-rich lipoproteins (including VLDL, intermediate-density lipoprotein, and chylomicron remnants) (60). In this study, the calculated VLDL encompasses VLDL, Intermediate-Density Lipoprotein (IDL),and chylomicron remnants, theoretically corresponding to RC (61). Observational and genetic studies (62, 63) have confirmed that elevated RC levels are associated with an increased risk of peripheral artery disease (PAD) (64), ischemic heart disease (65, 66), and ischemic stroke (67). Studies have also found that patients with higher RC levels still face an elevated risk of CAD even when receiving statin therapy (68). This study demonstrated that VLDL and VLDL/ApoB but not LDL, are associated with plaque stability, with elevated levels of VLDL and VLDL/ApoB serving as risk factors for the formation of unstable plaques. This suggests that in clinical practice, for patients with T2DM complicated by hypoechoic plaques, consideration should be given to using medications that target the reduction of VLDL and lower HDL/ApoA1 levels, in order to further reduce their residual risk of ASCVD.

Our study has some limitations. First, it is a retrospective cross-sectional single-center study, and some conclusions need to be verified in larger-scale, prospective, longitudinal, multicenter studies. Second, the RCS analysis showed no significant nonlinear associations between VLDL, VLDL/ApoB, HDL/ApoA1, and the grouping. Subsequent studies could further explore the relationships between these metrics and the grouping by expanding sample sizes and optimizing inclusion and exclusion criteria. Third, Statin therapy or antiplatelet treatment may have influenced the characteristics of atherosclerotic plaques. Recent evidence suggests that new oral hypoglycemic agents, GLP-1 RA or SGLT2i, have a modest anti-inflammatory and plaque-stabilizing effect (49).This study showed that statins, antiplatelet drugs, fibrates, metformin, and GLP-1 RAs/SGLT2 inhibitors had no significant effect on plaque characteristics. This finding may be associated with the small sample size of this study, as well as differences in medication doses and treatment courses. In the future, it is necessary to expand the sample size for further verification. Finally, in this study, standard ultrasound was used, and it has a variable diagnostic accuracy for specific components. A systematic review found that the highest diagnostic performance of standard US was for the detection of calcification (mean sensitivity 65.7%/mean specificity 84.7%), fibrous tissue (61.2%/84.9%), vulnerable/unstable plaque (76.3%/70.3%), and stable plaque (63.2%/82.7%) (69). In the future, advanced ultrasound technologies or other imaging modalities such as computed tomography (CT) and magnetic resonance imaging (MRI) can be adopted to improve the diagnostic performance for plaque components.

## 5 Conclusion

Overall, our study findings are valuable because they demonstrate that VLDL,VLDL/ApoB and HDL/ApoA1 are correlated with plaque stability, and theirs elevation are risk factors for the occurrence of unstable plaques. These findings suggest that, in clinical practice, the characteristics of atherosclerotic plaques (including echo types, structural features, and location distribution) and lipid profiles (including VLDL, VLDL/ApoB, HDL/ApoA1, and ApoA1/ApoB) should be evaluated in a combined manner. Meanwhile, attention should be paid to patients’ gender, age, hypertension, and duration of diabetes. Priority should be given to intervening in high-risk populations with hypoechoic plaques. For patients with type 2 diabetes complicated with hypoechoic plaques, medications that reduce VLDL and regulate HDL function may be considered to effectively lower the risk of atherosclerotic cardiovascular disease.

## Data Availability

The original contributions presented in the study are included in the article/supplementary material. Further inquiries can be directed to the corresponding author.
